# Isolation and near-complete genome of human enterovirus B4 (Coxsackie virus B4) strain isolated from a nasopharyngeal swab of a 5-year-old male child with severe hand, foot, and mouth disease in Mizoram, India

**DOI:** 10.1128/mra.00864-25

**Published:** 2025-11-10

**Authors:** Basavaraj Mathapati, Swagnik Roy, Vikas Sharma, Mallika Lavania, Souvik Mitra, Lalremruata Chenhrang, Dinesh Kumar Singh, Sanket Sonawane, Ratnadeep More, Naveen Kumar

**Affiliations:** 1 Basic Virology, ICMR-NIV East Zone, Dibrugarh, Assam, India; 2 Department of Microbiology, Zoram Medical College and Hospital 580765 , Falkawn, Mizoram, India; 3 Bioinformatics Group, ICMR-NIV 29620 , Pune, India; 4 Enterovirus Group, ICMR-NIV 29620 , Pune, India; 5 Polio Virus Group, ICMR-NIV 29620 , Pune, India; 6 Basic Virology, ICMR-NIV, Pune, Maharashtra, India; Katholieke Universiteit Leuven, Leuven, Belgium

**Keywords:** HFMD, CV B4, enterovirus

## Abstract

We report the near-complete genome sequence of Coxsackievirus B4 (CVB4) isolate BSM-25-005 (GenBank accession no. PX021639.1), collected in 2024 from a nasopharyngeal swab of a 5-year-old child with severe hand, foot, and mouth disease in Mizoram, India. Phylogenetic and sequence analyses identified the virus as CVB4 subgenotype D.

## ANNOUNCEMENT

Human enterovirus B4 (Coxsackievirus B4, CVB4) is one of the more than 150 enteroviruses in the genus *Enterovirus* of the family *Picornaviridae,* causing hand, foot, and mouth disease (HFMD) predominantly in children below 5 years of age (1). It causes acute infection with fever, mouth ulcers, and vesicular eruptions on hands, feet, and the ventral abdomen. It is responsible for several syndromes like myocarditis, meningoencephalitis, pleurodynia, hepatitis, pancreatitis, and respiratory illnesses. The infection can be severe, particularly in high-risk groups such as neonates and immunocompromised individuals (2, 3). HFMD is endemic in many parts of Asia (4) and has caused multiple outbreaks over the past decades. The BSM-25-005 strain of CVB4 was isolated from a nasopharyngeal swab of a 5-year-old male child with severe clinical manifestations of HFMD with existing Down Syndrome (trisomy 21) from Mizoram, India, in 2024. The clinical sample positive for a pan-enterovirus species-specific RT-PCR (4) was inoculated and isolated on Vero CCL-81 cells (5).

Viral RNA was extracted from the cell culture supernatant using the QIAamp Viral RNA Mini Kit (Qiagen, Germany), following the manufacturer’s protocol. Sequencing was performed using the Illumina Viral Surveillance Panel v2 (Illumina VSP v2 https://sapac.illumina.com/products/by-type/sequencing-kits/library-prep-kits/viral-surveillance-panel.html). Library preparation was done with the Illumina RNA Prep with Enrichment Kit (Illumina USA), in which libraries were enriched using VSP v2 probes through a hybrid-capture method, followed by on-bead tagmentation. The enriched libraries were then subjected to paired-end RNA sequencing on the Illumina NovaSeq 6000 platform (Illumina, USA). A total of 193,287 paired-end raw reads were quality-checked using FastQC (version 0.12.1). Low-quality sequences and adapters were trimmed using Fastp (version 0.23.4) (6).

**Fig 1:**
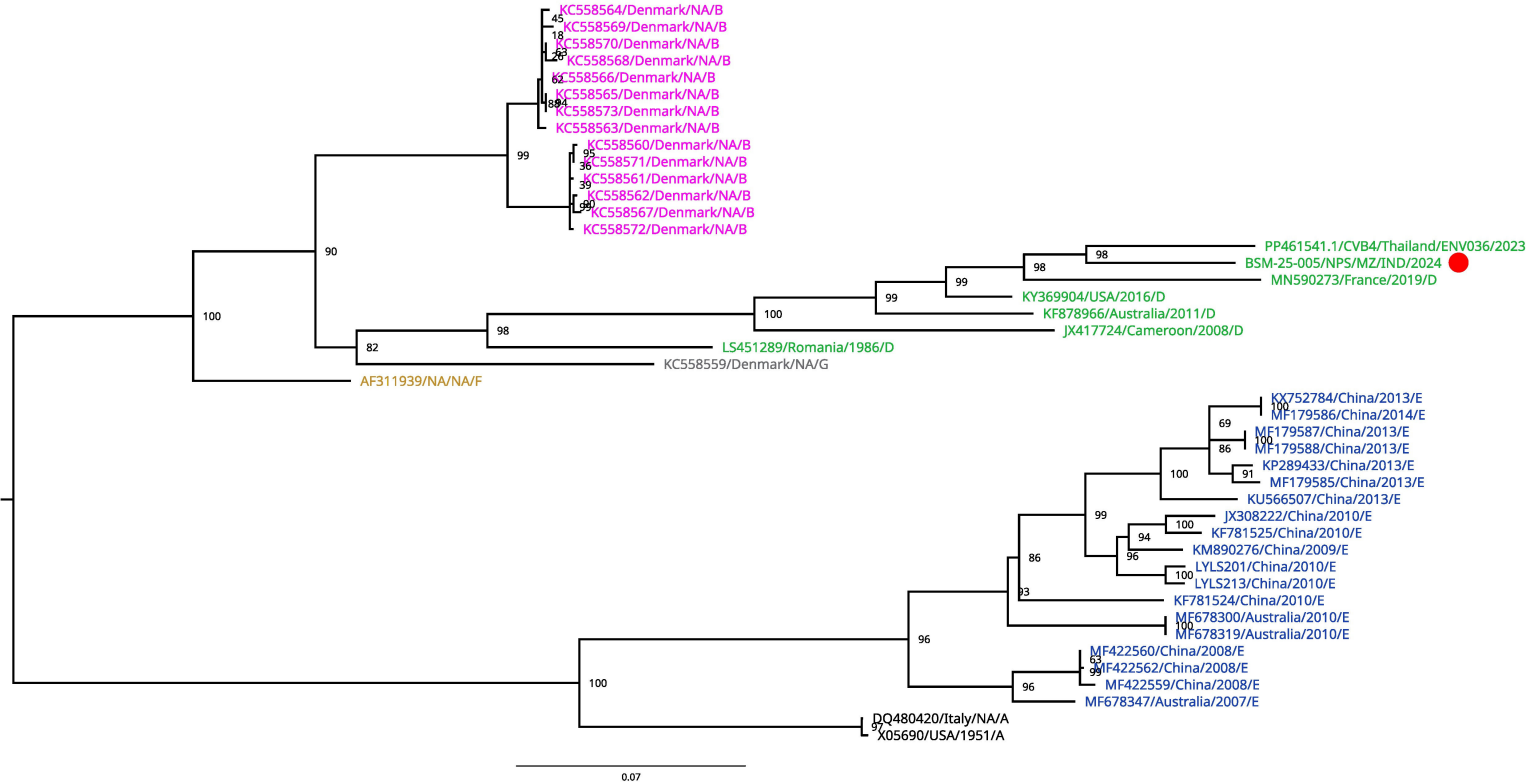
Multiple sequence alignment was performed using MAFFT version 7.526 with default parameters (11). ML phylogenetic tree of Coxsackievirus B4 (CVB4) was constructed based on the VP1 gene. The tree was inferred using IQ-TREE (version 2.0.7) (12) with the best-fit substitution model and 1,000 bootstrap replicates. Bootstrap values are shown at the corresponding branches. The tree was visualized using FigTree (http://tree.bio.ed.ac.uk/software/figtree/). Sequences are color-coded according to previously reported genotypes (A: black, B: pink, D: green, E: blue, G: gray, and F: brown). The CVB4 isolate from India is highlighted with a red circle.

The cleaned reads were assembled using two methods: *de novo* using rnaSPAdes (version 4.0.0) (7) and reference-based assembly through a custom pipeline. For reference-based assembly, reads were aligned to the reference genome (PP461541.1) using BWA-MEM (version 0.7.18) (8) and processed with Samtools (version 1.21) (9) for BAM conversion, sorting, indexing, and coverage calculation. Variant calling was performed with BCFtools (version 1.21), and a consensus genome was generated using bcftools consensus. The final assembled genome was 7,382 bp in length, with 47.4% GC content and an average depth of coverage of 2,572×. Although the assembled genome length (7,382 nt) matched that of the reference (PP461541.1), the genuine 5′ and 3′ termini could not be confirmed due to the absence of RACE; hence, the sequence is described as a near-complete genome. The assembled genome was annotated using VAPiD (version 1.6.7) (10), with default parameters against the RefSeq Viral Database (downloaded from NCBI FTP on 30 July 2024). The annotated genome was submitted to GenBank through the BankIt submission tool. Genotyping was performed using the Enterovirus Genotyping Tool (https://mpf.rivm.nl/mpf/typingtool/enterovirus/job/1599235367/), which identified the Indian isolated strain as CVB4. A BLASTn search against the NCBI NR database (accessed on 05 August 2025) revealed the closest match (88.59% nucleotide identity and 100% query coverage) to a CVB4 genome of Enterovirus B strain CVB4/Thailand/ENV036/2023 (PP461541.1) from Thailand, collected in 2023. An ML phylogenetic tree (Fig. 1) based on the VP1 gene confirmed this relatedness, placing the Indian CVB4 isolate within the CVB4 subgenotype D clade.

## Data Availability

The complete genome sequence of CVB4 has been submitted to GenBank via BankIt with the accession number PX021639. The corresponding raw sequencing reads have been deposited in the Sequence Read Archive (SRA) with the run accession PRJNA1301549 (Illumina RNA-seq of CVB4 from nasopharyngeal swab, Mizoram, India-SRA-NCBI).
